# Accuracy of Endoscopic Diagnosis of *Helicobacter pylori* Based on the Kyoto Classification of Gastritis: A Multicenter Study

**DOI:** 10.3389/fonc.2020.599218

**Published:** 2020-12-04

**Authors:** Jing Zhao, Shaoxian Xu, Yuan Gao, Yali Lei, Baicang Zou, Mimi Zhou, Danyan Chang, Lei Dong, Bin Qin

**Affiliations:** ^1^ Department of Gastroenterology, The Second Affiliated Hospital of Xi’an Jiaotong University, Xi’an, China; ^2^ Department of Gastroenterology, Shaanxi Provincial People’s Hospital, Xi’an, China; ^3^ Department of Gastroenterology, Ankang Central Hospital, Ankang, China; ^4^ Department of Gastroenterology, Weinan Central Hospital, Weinan, China

**Keywords:** unclear atrophy boundary, RAC reappearance in atrophic mucosa, early gastric cancer, *Helicobacter pylori*, Kyoto classification of gastritis

## Abstract

**Background:**

There is lack of clinical evidence supporting the value of the Kyoto classification of gastritis for the diagnosis of *Helicobacter pylori* (*H. pylori*) infection in Chinese patients, and there aren’t enough specific features for the endoscopic diagnosis of past infections, which is of special significance for the prevention of early gastric cancer (GC).

**Methods:**

This was a prospective and multicenter study with 650 Chinese patients. The *H. pylori* status and gastric mucosal features, including 17 characteristics based on the Kyoto classification and two newly-defined features unclear atrophy boundary (UAB) and RAC reappearance in atrophic mucosa (RAC reappearance) were recorded in a blind fashion. The clinical characteristics of the subjects were analyzed, and the diagnostic odds ratio (DOR), sensitivity, specificity, positive predictive value (PPV), negative predictive value (NPV), area under the receiver operating characteristics curve (ROC/AUC), and 95% confidence intervals were calculated for the different features, individually, and in combination.

**Results:**

For past infection, the DOR of UAB was 7.69 (95%CI:3.11−19.1), second only to map-like redness (7.78 (95%CI: 3.43−17.7)). RAC reappearance showed the highest ROC/AUC (0.583). In cases in which at least one of these three specific features of past infection was considered positive, the ROC/AUC reached 0.643. For current infection, nodularity showed the highest DOR (11.7 (95%CI: 2.65−51.2)), followed by diffuse redness (10.5 (95%CI: 4.87−22.6)). Mucosal swelling showed the highest ROC/AUC (0.726). Regular arrangement of collecting venules (RAC) was specific for no infection.

**Conclusions:**

This study provides evidence of the clinical accuracy and robustness of the Kyoto classification of gastritis for the diagnosis of *H. pylori* in Chinese patients, and confirms UAB and RAC reappearance partly supplement it for the diagnosis of past infections, which is of great benefit to the early prevention of GC.

## Introduction

Gastric cancer (GC) is a highly lethal malignancy, with only one in five patients surviving longer than five years after diagnosis (1). Most gastric adenocarcinomas, particularly those of the intestinal type, are associated with a sequence of phenotypic changes of the native mucosa triggered by long-standing inflammation, induced mostly by *H. pylori* (2). Approximately 89% of all gastric cancers can be attributed to *H. pylori* infection. It has been reported that 14.2% of gastric cancers occur in patients with past *H. pylori* infections, while only 0.42% of gastric cancers occur in uninfected patients (3). Therefore, clarifying the *H. pylori* infection status of patients is of great importance for the detection of early GC.

Different invasive and non-invasive tests are available to detect *H. pylori* infection. Invasive methods are based on gastric biopsy samples and include *H. pylori* culture, histological staining, rapid urease test (RUT) and PCR methods. Non-invasive methods include the urease breath test, *H. pylori* stool antigen test and serum IgG tests (4). The accuracy of the invasive methods is affected by inevitable external factors, like the location, size, and quantity of biopsy samples, the staining method, use of proton pump inhibitors, antibiotic administration, and experience of the examiners (5). Non-invasive examinations are cheap, fast, and easy to perform, but there are also factors that can affect their diagnostic accuracy, such as the use of antibiotics, bismuth agents, some traditional Chinese medicines for the treatment of *H. pylori*, as well as the test reaction time (6).

*H. pylori* infection triggers inflammation, and its eradication diminishes inflammation, which is manifested histologically as aggregation, infiltration, and disappearance of multinuclear and mononuclear cells, destructing and restoring the microstructure of the gastric mucosa. Increasingly advanced endoscopic techniques have made it possible to visualize minute mucosal structures, such as the patterns of gastric pits and microvascular branching, raising the possibility of diagnosing *H. pylori* infection by endoscopy (7).

Conventional endoscopy, the most widely used endoscopic technique, was thought to be a poor method to diagnose the *H. pylori* status, since *H. pylori* gastritis does not produce specific manifestations detectable under conventional endoscopy, and infection is usually distributed in multiple foci (8, 9). However, this view changed when the Kyoto classification was published in Japan in 2014. This classification permits the diagnosis of *H. pylori* gastritis and an evaluation of gastric cancer risk under endoscopic examination (10). Nevertheless, endoscopic features may differ based on the geographic location and the ethnicity of patients. For example, some features which are typical of GC in Asian patients may not be present in Caucasian patients (11). It has been reported that there are significant differences in gastric mucosa of gastric cancer patients from different countries and regions in Asia. Therefore, endoscopic features associated with the *H. pylori* status may also differ between Chinese and Japanese patients, despite the high incidence of GC in both populations. This indicates that more evidence is needed to conclude that the Kyoto classification-based conventional endoscopic features are clinically effective for determining the *H. pylori* status in different populations. Moreover, there are rare specific signs of past infection in the Kyoto classification, making it difficult to distinguish these cases from uninfected patients. As mentioned earlier, patients with past *H. pylori* infection and uninfected ones have a different risk of GC. Hence, another aim of this study was to clarify the usefulness of two new features, “unclear atrophy boundary (UAB)” and “RAC reappearance in atrophic mucosa (RAC reappearance)”, for the diagnosis of past infections. These signs were first noticed in patients with past infections in our clinical practice and have not been studied before.

## Materials and Methods

### Subjects

This was a prospective, multicenter study, in which four facilities (the Second Affiliated Hospital of Xi’an Jiaotong University, Shaanxi Provincial People’s Hospital, Ankang Central Hospital and Weinan Central Hospital) participated. A total of 650 patients >18 years old who had undergone upper gastrointestinal endoscopy) were consecutively recruited in the four facilities between July 2018 and December 2019 (202 in the Second Affiliated Hospital of Xi’an Jiaotong University, 120 in the Shaanxi Provincial People’s Hospital, 157 in the Ankang Central Hospital and 171 in the Weinan Central Hospital). The exclusion criteria were as follows: severe brain, liver, or cardiopulmonary dysfunction, end-stage renal disease requiring dialysis, schizophrenia, or other mental diseases interfering with patient cooperation, pregnancy, patients with pyloric obstruction or poor preparation (who had to withdraw due to excessive food residue interfering with the endoscopy), treatment with antibiotics or proton pump inhibitors (PPIs) four weeks prior to study initiation, previous diagnosis of early or advanced gastric cancer, gastrectomy, or hemorrhagic tendency.

Assuming 80% sensitivity/specificity, the required sample size was 264 to keep the 95% confidence interval within ±5%. If the prevalence of *H. pylori* is 50% (estimated at 55.8% in China (1)), the total sample size needed to be 528. We finally set the final sample size at 581, taking into consideration the possibility of incomplete or incorrect data in 10% of the subjects.

This study was approved by the ethics committee of The Second Affiliated Hospital of Xi’an Jiaotong University (Ethics approval No.2018076). All participating subjects signed an informed consent.

### Procedures

In this study we investigated the association between endoscopic features and a positive diagnosis of *H. pylori* infection made by traditional detection methods (urease breath test and rapid urease test), as well as the patient’s past history. Blindness method was used to collect data, and control information bias. The endoscopic examiner was blinded to the *H. pylori* test results and to the past history of patients, which were both accurately recorded by a separate investigator before the endoscopy. The primary endpoint was the diagnostic value of each endoscopic feature for *H. pylori* infection, determined individually. The secondary endpoint was the diagnostic value of endoscopic features for *H. pylori* infection, determined in combination.

### Diagnosis of *H. pylori* Infection

A specific interviewer was responsible for recording the patients’ responses to an inquiry of past history of *H. pylori* infection at each facility. Every patient was required to undergo at least one of the diagnostic tests [urease breath test (13C-UBT or 14C-UBT) or rapid urease test (RUT)], within two weeks of the gastroscopy, and these results were also recorded by the interviewer.

The following methods and equipment were used to determine the *H. pylori* status of the participants: HY-IREXB *Helicobacter pylori* detector (Guangzhou Huayou Mingkang Photoelectric Technology Co., Ltd.), urea [13C] breath test diagnostic kit (Beijing Huabo Medical Technology Co., Ltd.); YH04F *H. pylori* detector, YH04 series *H. pylori* breath card (Anhui Yanghe Medical Equipment Co., Ltd.), 14C capsule (Shanghai Xinke Pharmaceutical Co., Ltd.); and *Helicobacter pylori* rapid detection test (Guangzhou beisiqi Diagnostic Reagent Co., Ltd.) for rapid urease test.

Based on the above results, the *H. pylori* status of the participants was divided into the following three types: 1) “Past infection” (eradicated): When more than four weeks had elapsed after a single and only *H. pylori* eradication event, subjects who were currently confirmed negative by either RUT or 13C-UBT/14C-UBT tests. 2) “No infection”: Subjects without a history of *H. pylori* eradication who were confirmed negative by any of the three testing methods. 3) “Current infection”: Subjects without a history of eradication who were confirmed positive by any of the three methods.

The endoscopists were blinded to the *H. pylori* status of each subject before and during the operation.

### Endoscopic Assessment of Different Features

Five endoscopists performed endoscopy in the study (two in the Second Affiliated Hospital of Xi’an Jiaotong University and one in each of the other three centers). To improve diagnostic accuracy among participating facilities, all endoscopists were experienced, having performed over 5,000 gastroscopies and were familiar with the Kyoto classification of gastritis after twice pre-study training. In order to obtain uniform endoscopic diagnoses and to avoid inter-operator variability, abstracts summarizing typical images of endoscopic features were distributed to each endoscopist before study initiation.

All procedures were performed by well-trained endoscopists using high-resolution electronic endoscopes (GIF-HQ 260, Olympus Medical Systems) which allowed clear visualization of the collecting venules. Oxybuprocaine hydrochloride gel (30 mg, Shenyang Oasis Pharmaceutical Co., Ltd, China) and dimethylsilicone oil powder (0.5−1%, Jianheng, Zigong Honghe Pharmaceutical Co., Ltd) were used before and during endoscopy.

**Figure 1:**
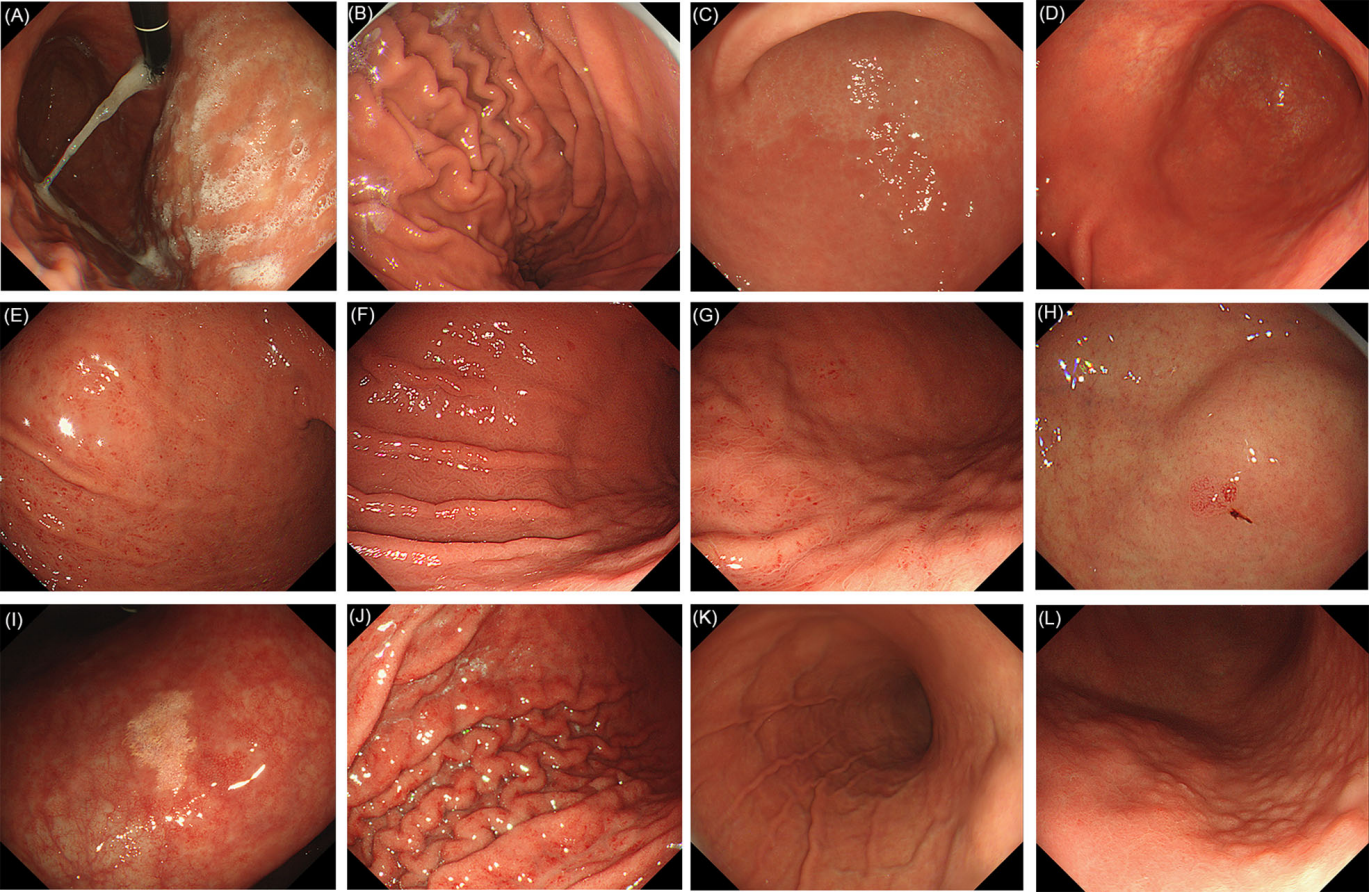
Evaluated endoscopic features for *H. pylori* current infection. **(A)** Sticky mucus: present. **(B)** Sticky mucus: absent. **(C)** Atrophy. **(D)** Diffuse redness. **(E)** Spotty redness. **(F)** Mucosal swelling. **(G)** Spotty redness, along with mucosal swelling. **(H)** Hyperplastic polyp. **(I)** Xanthoma. **(J)** Enlarged fold/tortuous fold: present. **(K)** Enlarged fold/tortuous fold: absent. **(L)** Nodularity.

**Figure 2:**
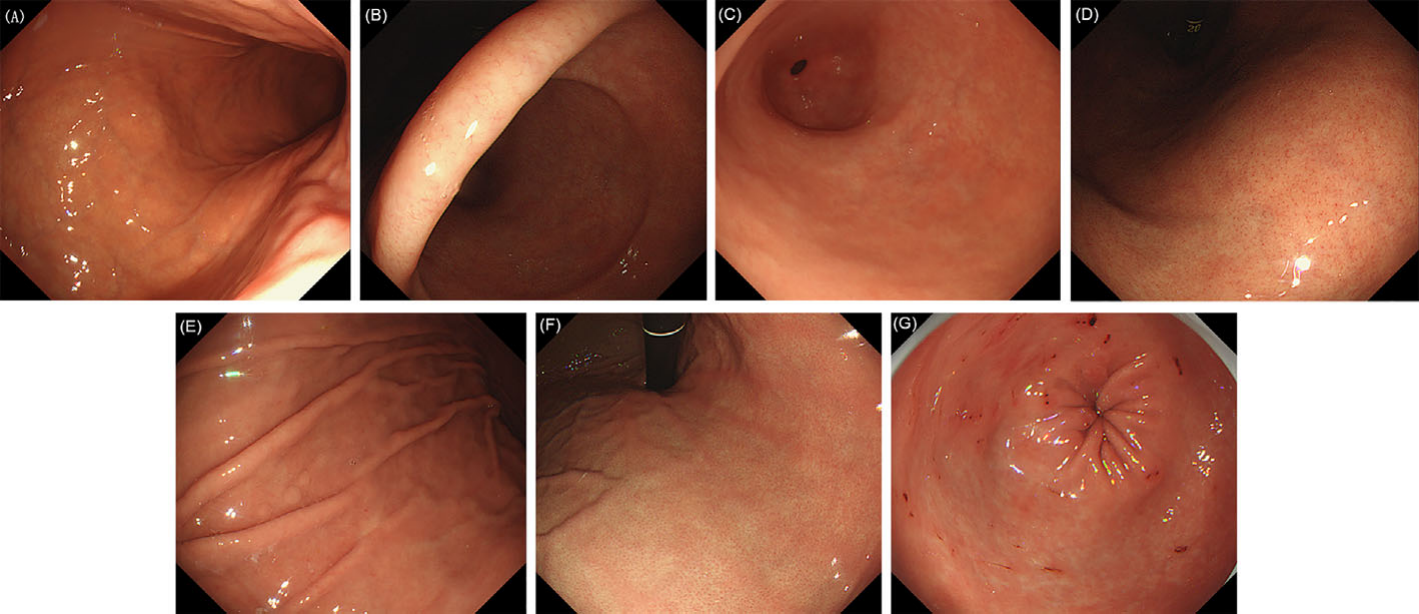
Evaluated endoscopic features for *H. pylori* no infection. **(A)** Normal mucosa of gastric corpus. **(B)** Normal mucosa of gastric gastric angle. **(C)** Normal mucosa of gastric antrum. **(D)** Regular arrangement of collecting venules (RAC). **(E)** Fundic gland polyp (FGP). **(F)** Red streak. **(G)** Hematin.

**Figure 3:**
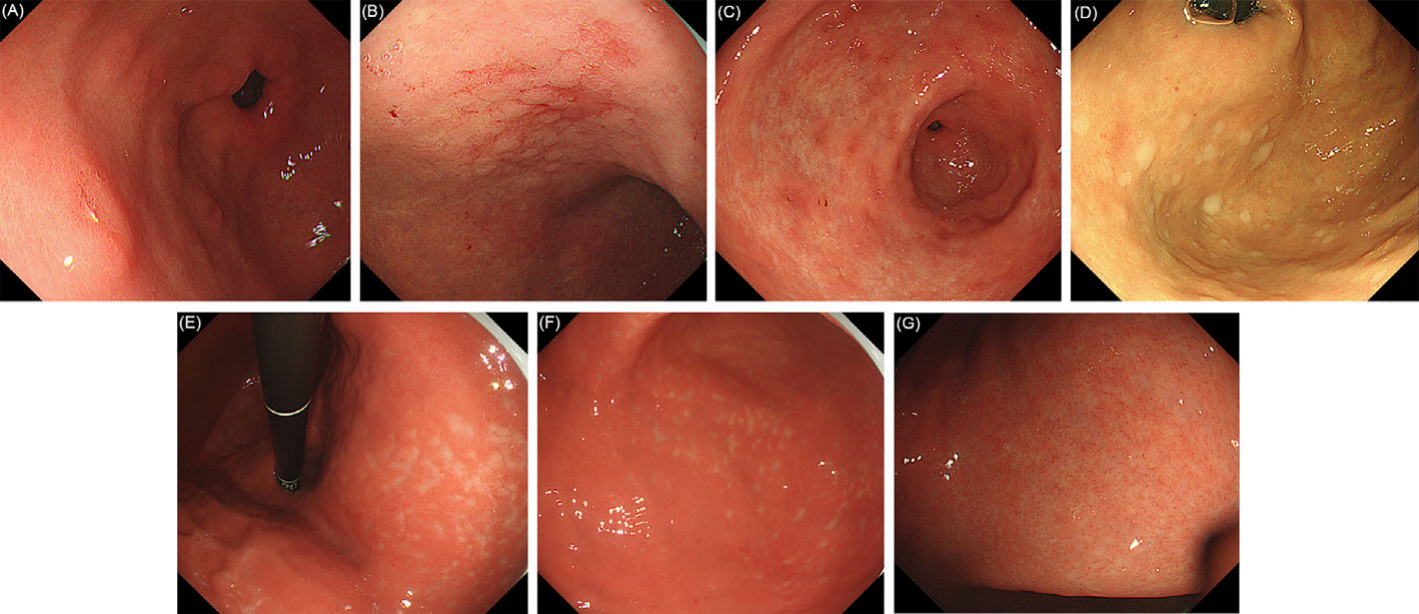
Evaluated endoscopic features for *H. pylori* past infection. **(A)** Raised erosion. **(B)** Map-like redness of gastric corpus. **(C)** Map-like redness of gastric antrum. **(D)** Multiple white and flat elevated lesions. **(E)** Unclear atrophy boundary in lesser curvature of the stomach. **(F)** Unclear atrophy boundary in greater curvature of the stomach. **(G)** RAC reappearance in atrophic mucosa after *H. pylori* eradication.

The following 17 distinctive endoscopic features related to *H. pylori* status (uninfected, infected, or eradicated) were defined based mainly on the Kyoto classification of gastritis (10): 1) sticky mucus, 2) atrophy, 3) diffuse redness, 4) spotty redness, 5) mucosal swelling, 6) hyperplastic polyp, 7) xanthoma, 8) enlarged fold/tortuous fold, 9) nodularity, 10) regular arrangement of collecting venules (RAC), 11) fundic gland polyp (FGP), 12) red streak, 13) hematin, 14) raised erosion, 15) map-like redness, 16) cobblestone-like mucosa, and 17) multiple white and flat elevated lesions. In addition, UAB was defined as atrophy without a clear line of separation between redness and whiteness, but with a spot-like appearance instead. RAC reappearance was defined as reappearance of typical or atypical RAC in atrophic gastric mucosa. Patients with both atrophy (graded C2 or higher), and RAC were defined as positive for “RAC reappearance”. These features were divided into three categories (10), as follows: 1−9) are reported to be strongly associated with current infection with *H. pylori*, 10−13) with non-infection and 14−17) plus the new features (UAB and RAC reappearance) with past infection. Because of the multiple diagnostic significance of some features, the diagnostic odds ratios for each feature were calculated in relation to the three *H. pylori* states, as a supplement to the Kyoto classification of gastritis. Based on the results, the features were further classified into categories defined by their highest diagnostic tendency, and on this basis further statistical analyses were carried out. Intestinal metaplasia in the Kyoto classification of gastritis was not included because it itself is difficult to be accurately diagnosed by conventional endoscopy. Hence, it was usually considered as a histological diagnosis rather than an endoscopic diagnosis in clinics in China. Typical endoscopic images are shown in **Figures 1**–**3**. The presence or absence of each feature was evaluated during the endoscopy based on the diagnostic criteria. Immediately after the examination, the endoscopist recorded whether the features were present or not.

### Quality Control

To ensure the authenticity and validity of the statistical analyses, we designed the recording form with opposing groups, like “RAC present” and “RAC absent”. Forms in which none of the items were selected were considered invalid. In addition, we also included a supplementary group classification, with categories like “atrophy” and “UAB”. Forms in which “UAB” was selected but “atrophy” was not were likewise considered invalid.

### Statistical Analysis

The diagnostic odds ratios (DORs) and 95% confidence intervals of the endoscopic features for the three *H. pylori* status were calculated. One-way ANOVA was used to distinguish age differences between the three different *H. pylori* status groups. Chi-squared test was used to analyze gender and features differences. *P* < 0.05 was regarded as significant. Sensitivity, specificity, PPV, NPV, ROC/AUC, and 95% confidence intervals were calculated for those features showing significant statistical differences. All statistical analyses were performed using IBM SPSS Statistics 21.

## Results

### Patient Characteristics

**Figure 4:**
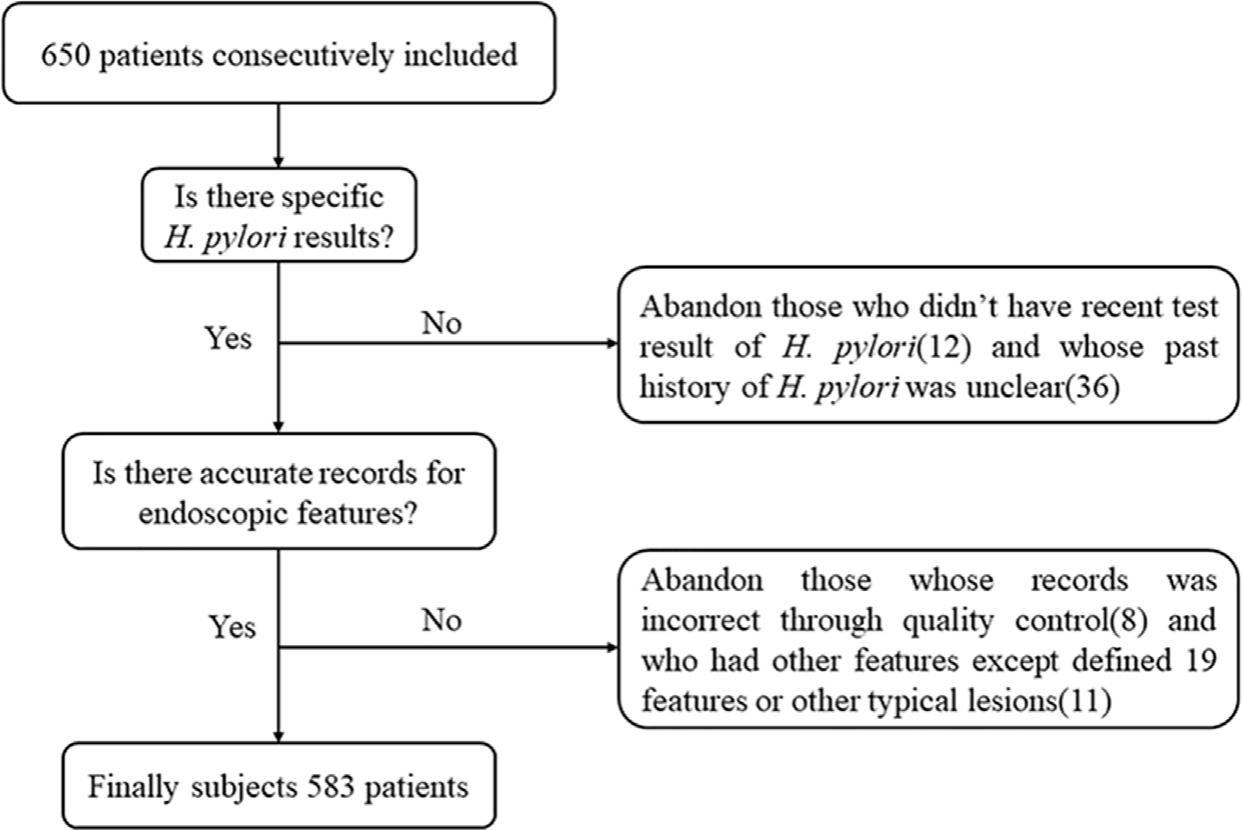
Schematic view of subjects screening.

A total of 650 patients were consecutively recruited. Those who didn’t have a recent *H. pylori* test result (n = 12) and those with an unclear past history of *H. pylori* infection (n = 36) were excluded. Next, we verified whether there were accurate records of the endoscopic features we defined. A total of 583 patients were finally included in the study, after excluding those with incorrect records based on quality control (n = 8) and those who had other characteristics in addition to the 19 defined features, or other typical lesions such as gastric ulcer, early GC or other (n = 11) (**Figure 4**).

Regarding the baseline characteristics of the patients, 226 (38.8%) were classified as “no infection” patients, 246 (42.2%) as “current infection” patients, and 111 (19.0%) as “past infection” patients. Their mean ages (SD) were: 47.9 (13.4), 45.9 (13.9), and 49.1 (13.6) years, respectively, with no significant difference between groups (*P* = 0.082). Among 278 (47.7%) male patients, 95 (34.2%) were classified as “no infection”, 116 (41.7%) as “current infection” and 67 (24.1%) as “past infection”. Among 305 (52.3%) female patients, the corresponding numbers were 131 (43.0%), 130 (42.6%), and 44 (14.4%), respectively. No infection and past infection patients showed significant difference in terms of gender (*P* = 0.007).

18 features were further analyzed (excluding cobblestone-like mucosa, which was not observed in any of the subjects). The DORs of the individual endoscopic features for the three *H. pylori* status are shown in **Table 1** (**Supplementary Material**). In contrast to the overwhelming majority of features which showed the same diagnostic tendency (as expected), atrophy showed the same DOR (1.91) for current infection and past infection. In addition, the DOR of RAC for no infection was 4.64, and for past infection 1.74. As expected, the DOR of UAB for past infection was as high as 7.69, second only to map-like redness (7.78), and its DOR for current infection was 0.137, meaning that it was unlikely to be present in current infection cases. Another newly defined sign, RAC reappearance, showed similar diagnostic efficacy.

### Diagnostic Efficacy of Features for Current Infection

Associations between endoscopic features and current *H. pylori* infection are shown in **Table 2** (**Supplementary Material**). Among the nine features which are supposed to suggest “current infection”, atrophy, mucosal swelling and spotty redness appeared most frequently, in 266/583 (45.6%), 203/583 (34.8%), and 184/583 (31.6%) of the cases, respectively. The frequency of the other features was less than 10% of the total.

Regarding single features, sticky mucus, atrophy, diffuse redness, spotty redness, mucosal swelling and nodularity showed significant differences between “current infection” and the other two groups (“no infection” and “past infection”). The ROC/AUCs of the first five showed statistical significance, but this was not true in the case of nodularity. Mucosal swelling showed the highest ROC/AUC (0.726), and its sensitivity (61.0%) and NPV (74.7%) were also the highest. On the other hand, nodularity showed the highest specificity (99.4%) and PPV (88.9%).

When the nine individual signs were analyzed together, the ROC/AUC of only one positive feature wasn’t statistically significant. The ROC/AUC of two or more positive features showed the highest value (0.723), followed by one or more, in which case the sensitivity and NPV showed the highest values (94.3 and 91.3%, respectively). According to the DOR value, there was a close relationship between nodularity, diffuse redness, mucosal swelling and “current infection” among the single features. When cases testing positive for at least one of the three previously mentioned endoscopic findings were classified as current infection, the sensitivity was 69.1%, the specificity was 82.5%, the PPV was 74.2%, the NPV was 78.5%, and the ROC/AUC was 0.758 (95% CI:0.717–0.799); these were the highest values for all single and combined features. When cases which were positive for at least two of these features were classified as “current infection”, the sensitivity was 17.1% and the specificity was 98.8%. When the number reached three, the sensitivity and specificity were 1.63% and 100%, respectively.

### Diagnostic Efficacy of Features for No Infection

Among the four features suggestive of “no infection” (**Table 3**, **Supplementary Material**), RAC and hematin were most frequently observed, in 235/583 (40.3%) and 80/583 (13.7%) of the cases, respectively, while the rest were below 10% of the total. RAC, red streak, and hematin showed significant differences between “no infection” and the other two groups (“current infection” and “past infection”), with all three showing statistically significant ROC/AUCs. RAC showed the highest ROC/AUC (0.680), the highest sensitivity (62.4%), and the highest NPV (75.6%), but the lowest specificity (73.7%).

Regarding combined features, one or more positive features showed the highest ROC/AUC (0.701), the highest sensitivity (94.3%), and the highest NPV (91.3%). When cases testing positive for at least three features were classified as “no infection”, the specificity and PPV showed the highest values (99.7 and 94.4%, respectively).

### Diagnostic Efficacy of Features for Past Infection

Six features for “past infection” were included in the study, including atrophy (**Table 4**, **Supplementary Material**). The frequency of these signs was low, with the exception of atrophy (n = 266). RAC reappearance (n = 58), came after atrophy, representing only 9.95% of the total.

RAC reappearance, atrophy, map-like redness, UAB, and raised erosion showed significant differences between “past infection” and the other two groups (“current infection” and “no-infection”). The ROC/AUCs of the first three features showed statistical significance, but this was not the case for the last two. Among all the single features, RAC reappearance showed the highest ROC/AUC (0.583, 95%CI: 0.520−0.646). UAB showed the highest PPV (61.9%) and the second highest specificity (98.3%). However, it was observed in only 21 patients (3.60%) and its ROC/AUC was low (0.550, 95% CI: 0.488−0.613), making it difficult to evaluate in this study. The sensitivity and NPV of atrophy showed the highest values (58.6 and 85.5%, respectively).

The results of the combined analysis of these six signs are as follows: When the number of combined positive features was increased (from one or more features to three or more), the sensitivity and NPV decreased, while the specificity and PPV increased. In general, when cases testing positive for at least two features were classified as “past infection”, the ROC/AUC was the highest (0.617). The distribution and diagnostic performance of atrophy combined with UAB were the same as that of UAB alone, since atrophy necessarily had to be present when UAB was detected. In addition, the ROC/AUC of either map-like redness positive or atrophy positive was 0.597 (95%CI: 0.539−0.655), while its sensitivity and specificity were 62.2 and 57.2%, respectively. When cases testing positive for at least one of the three features (map-like redness, UAB or RAC reappearance) were classified as past infection, the sensitivity was 37.8%, the specificity was 90.7%, the PPV was 48.8%, the NPV was 86.1%, and the ROC/AUC was 0.643 (95%CI: 0.580–0.705); these were the highest values in the analysis for all single and combined features of past infection. Moreover, the ROC/AUC of either UAB positive or RAC reappearance positive was 0.614 (95%CI: 0.551−0.677), higher than any other single feature.

## Discussion

*H. pylori* has been identified as a Group I carcinogen by the International Agency for Research on Cancer. Timely endoscopic identification of current infection and past infection is of great benefit to the monitoring of high risk population of early GC. This was a prospective, multicenter study to evaluate the diagnostic value of endoscopic features for *H. pylori* infection status, mainly based on the Kyoto classification of gastritis. In addition, two new features, “UAB” and “RAC reappearance”, were investigated, which were beneficial for the diagnosis of past infection.

We divided the *H. pylori* status of the participants into three categories: *H. pylori* positive (“current infection”), *H. pylori* negative (“no infection”) and *H. pylori* negative after eradication (“past infection”). Of note, the risk of GC is far greater in patients who are *H. pylori* negative after successful eradication than in uninfected patients (3). Traditional testing methods such as the urease breath test, rapid urease test, and others cannot make a direct, accurate diagnosis of “past infection”. Hence, in cases in which the patient’s past history is unknown, it would be clinically important to be able to determine past infection endoscopically. Importantly, the *H. pylori* status and endoscopic features were recorded separately in this study, avoiding subjective influence. Additionally, cooperation between multiple centers improved the comprehensiveness and integrity of the data.

Swelling and redness of the gastric mucosa have been endoscopically confirmed in cases with *H. pylori* -induced inflammation (12, 13). Mucosal swelling has become the most valuable feature for the diagnosis of *H. pylori*-infection of the gastric mucosa, with a ROC of 0.726 in published studies. Nowadays, high-resolution endoscopy permits the detection of mucosal unevenness and swelling of the *areae gastricae*, even without the use of the indigo carmine contrast (IC) method, as in former studies (14). Diffuse redness is considered to be a marker of histologic mucosal hyperemia, and this feature strongly associates with the hemoglobin index (IHb), an objective index of redness (12). In Kato’s study, it was concluded that diffuse redness was a diagnostically useful endoscopic finding in *H*. *pylori*-infected stomachs (14). Consistent with previous studies, we found diffuse redness to be highly associated with current *H. pylori* infection, with a ROC/AUC of 0.590 (13, 14). Nodularity is the result of lymphofollicular hyperplasia, and it is considered strong evidence in favor of *H. pylori* infection (15–17). Our results are in agreement with these conclusions since nodularity showed the highest DOR (11.7), although its ROC/AUC was low because of its low frequency (only observed in 18 patients). Based on the DORs, we found there was a close relationship between nodularity, diffuse redness, mucosal swelling and “current infection” among the single features. When cases testing positive for at least one of these three endoscopic features were classified as “current infection”, the ROC/AUC was 0.758, which was the highest in the study, underscoring the importance of paying attention to these three features during evaluation.

On the other hand, consistent with previous studies, sticky mucus, atrophy, spotty redness, hyperplastic polyp, xanthoma and enlarged/tortuous folds were suggestive of current *H. pylori* infection (14, 18, 19). Considering all nine features, if only one is present, it is insufficient to diagnose a current *H. pylori* infection (see **Table 2**). But when two or more are positive, all these features are useful for evaluation (ROC/AUC 0.723). Early detection of *H. pylori* gastritis and prompt eradication are an effective therapeutic strategy for the prevention of gastric cancer (20, 21). From this point of view, our results are promising, contributing to the early detection of *H. pylori* gastritis.

In contrast, RAC, red streak, hematin, and FGP have been reported to be correlated with an *H. pylori*-negative, normal stomach (14, 18, 19). In accordance with previous studies, RAC showed a good diagnostic value for non-infected patients in this study, with a ROC of 0.680 (22). Consistent with previous studies, the other three features also showed a certain diagnostic value, but their frequency was low, so two or more seldom appeared simultaneously. Cases positive for one or more of these features and diagnosed as “no infection” showed the highest ROC/AUC (0.701). Undoubtedly, the more these features are present simultaneously, the more likely the gastric mucosa will be *H. pylori* negative.

Successful eradication of *H. pylori* improves gastritis and may prevent various diseases associated with *H. pylori* infection (23). The diagnosis of past *H. pylori* infection is especially important for the early monitoring of gastric cancer. It is well documented that *H. pylori* eradication alleviates histologic gastritis (24). In terms of histological parameters, most studies report similar trends, such as disappearance or reduction of inflammatory cells, including both polymorphonuclear cells and mononuclear cells (24). However, due to a lack of specific endoscopic signs, previous studies usually diagnosed *H. pylori* eradication based on an improvement of signs of “current infection”. For example, Kato et al. found that regression of spotty redness after eradication suggested past infection (25). Using magnifying endoscopy, Yagi et al. concluded that mucosal swelling disappeared and mucosal redness improved after eradication (26). However, strict and continuous endoscopic monitoring of the gastric mucosa before and after *H. pylori* eradication is too difficult and expensive to achieve in real clinical situations in China. In this regard, map-like redness as well as cobblestone-like mucosa have been reported to be specific for past infection in the Kyoto gastritis classification, and this undoubtedly constitutes a great breakthrough in endoscopic diagnosis (18, 27, 28).

No cobblestone-like mucosa was seen in all cases in this study. However, map-like redness was indeed an effective diagnostic index, with a DOR of 7.78 (which was the highest) and a significant ROC/AUC of 0.561. But more important were the other two specific features investigated in this study, UAB and RAC reappearance. Previous long-term follow-up studies of patients showed that the degree of atrophy of the gastric mucosa can be reduced after *H. pylori* eradication, but whether the atrophic boundary becomes blurred due to the disappearance of inflammation has not been determined (29). We detected and defined this finding as “unclear atrophy border (UAB)”. In this study, UAB was highly correlated with past infection (DOR 7.69). Its sensitivity was low (11.7%), but its specificity (98.3%) was very high and its accuracy similar to that of map-like redness (81.8%). However, low frequency prevented an accurate evaluation of its diagnostic efficacy. More samples and renewed focus on this new specific feature are warranted. Moreover, RAC reappearance, which has often been ignored in previous traditional endoscopic studies, also effectively indicated past *H. pylori* infection, a finding that is in agreement with the results of Yagi et al. using magnifying endoscopy (26). *H. pylori*-infected and inflamed gastric mucosa, characterized by the continuous breakdown and regeneration of blood vessels due to severe inflammation, will show remarkable changes in these gastric mucosal patterns if successfully treated (24, 30). The density of fine irregular vessels will decrease, and RAC may reappear, even in atrophic mucosa resulting from persistent inflammation. Although it was observed in only 21 cases in this study, its specificity was 93.2% and its ROC/AUC (0.583) was the highest for a single sign. Hence, this feature will be of great benefit for the diagnosis of past infection. When cases testing positive for at least one of these two features (UAB and RAC reappearance) were classified as past infection, the ROC/AUC was 0.614, suggesting that these two features may be unique indicators of past *H. pylori* infection in Chinese patients. When cases testing positive for at least one of these two features or map-like redness were classified as past infection, the ROC/AUC reached 0.643, which was the highest score, a rare finding, since all of these features of “past infection” are uncommon.

Atrophy, which is caused by *H. pylori* infection, is certainly observed in the gastric mucosa with current infection (31). After eradication, the atrophy improves in degree, but usually still persists, even if the boundary becomes unclear (29). Because it is present in patients in whom *H. pylori* has been eradicated, atrophy is not specific for either current infection or past infection. As in previous studies, raised erosion and multiple white and flat elevated lesions suggested past *H. pylori* infection to some extent (10). When analyzing these six features together, the ROC/AUC was a significant 0.617, highlighting the importance of paying attention to all these features during evaluation.

For the diagnosis of *H. pylori* status, it is first necessary to assess the presence or absence of atrophy. This was the most common feature in nearly half of the samples. The presence of atrophy is rarely indicative of “no infection”. Therefore, if it occurs, further careful observation of whether there is RAC reappearance in the atrophic background or unclear atrophy boundary is the key to determine whether it corresponds to “past infection”. With the exception of atrophy, features of past infection like these two usually appear with low frequency but have high specificity; that is, once any appears, the diagnostic accuracy for determining past infection is very high. On the contrary, if there is no such feature, but mucosal swelling, diffuse redness, and other signs appear together with atrophy, then the diagnosis of “current infection” is more likely. In general, a single feature indicative of current infection can achieve relatively ordinary diagnostic value, but the more features, the higher the accuracy. On the other hand, if the patient does not have atrophy, it is very likely that he has never been infected with *H. pylori* (“no infection”). If specific features of no infection such as RAC, red streak, and others can also be observed, “no infection” can be diagnosed. The probability of two or three kinds of correlating features appearing at the same time is small, so judgment is usually made according to the features which appear more frequently. On the other hand, it is also possible to combine the patient’s past history to assist in the diagnosis and even return to the traditional methods like urease breath test, rapid urease test and others to diagnose the more difficult cases. After all, the ultimate goal is to reach the best clinical diagnosis.

We acknowledge that our study has some limitations. First of all, although abundant time was devoted to studying the Kyoto classification of gastritis and each feature was defined uniformly and strictly before study initiation, the assessment of these features depended on the endoscopists themselves during examination, so there may have been some inter- and intra-observer variability. Second, natural elimination of *H. pylori* infection or unintentional *H. pylori* eradication may have been underestimated; that is, patients with no history of eradication and negative test results were classified as “no infection” according to the classification, but features like UAB, RAC reappearance and so on may have appeared due to “past infection”; third, traditional detection methods, considered the gold standard in this study, may have produced false negative or false positive results, leading to some degree of error in the actual classification of *H. pylori* status.

In conclusion, this is the first study that provides evidence of the clinical accuracy and robustness of the Kyoto classification of gastritis in the Chinese population and provides two new indicators of past *H. pylori* infection, UAB and RAC reappearance in atrophic mucosa as the supplement, giving the judgment of *H. pylori* sufficient endoscopic basis. We believe that, despite its limitations, our study offers important new findings for screening of early GC based on the close relationship between *H. pylori* and GC.

## Data Availability Statement

The raw data supporting the conclusions of this article will be made available by the authors, without undue reservation.

## Ethics Statement

The studies involving human participants were reviewed and approved by the ethics committee of the Second Affiliated Hospital of Xi’an Jiaotong University. The patients/participants provided their written informed consent to participate in this study.

## Author Contributions

JZ, BQ, and LD designed the study. JZ, BQ, SX, YG, YL, and BZ collected and analyzed the data. JZ drafted the manuscript. LD, MZ, and DC revised the manuscript. All authors contributed to the article and approved the submitted version.

## Funding

This work was supported by the 2016 Special Fund Project for Local Science and Technology Development Guided by the Central Government of China [2016ZY-HM-01], and the Fundamental Research Funds of Xi’an Jiaotong University.

## Conflict of Interest

The authors declare that the research was conducted in the absence of any commercial or financial relationships that could be construed as a potential conflict of interest.
