# Supplementary material for: Accuracy of Endoscopic Diagnosis of Helicobacter pylori Based on the Kyoto Classification of Gastritis: A Multicenter Study
Source: Front Oncol. 2020 Dec 4;10:599218. doi: 10.3389/fonc.2020.599218 (PMC7746828; doi:10.3389/fonc.2020.599218)
Supplement: Supplementary file 1 [file DataSheet_1.doc]

Supplementary Material

# Supplementary Tables

**Table 1. Diagnostic odds ratio of the individual endoscopic features**

|  | **Current infection** | **No infection** | **Past infection** |
| --- | --- | --- | --- |
| **Sticky mucus** | **4.29*** | 0.446* | 0.142* |
| (95% CI) | (2.32-7.96) | (0.234-0.849) | (0.034-0.592) |
| **Atrophy** | **1.91*** | 0.324* | **1.91*** |
| (95% CI) | (1.37-2.67) | (0.227-0.462) | (1.25-2.90) |
| **Diffuse redness** | **10.5*** | 0.101* | 0.289* |
| (95% CI) | (4.87-22.6) | (0.036-0.283) | (0.103-0.817) |
| **Spotty redness** | **2.69*** | 0.455* | 0.645 |
| (95% CI) | (1.88-3.85) | (0.311-0.667) | (0.401-1.04) |
| **Mucosal swelling** | **8.37*** | 0.171* | 0.397* |
| (95% CI) | (5.67-12.4) | (0.111-0.263) | (0.241-0.657) |
| **Hyperplastic polyp** | **2.63*** | 0.516 | 0.463 |
| (95% CI) | (1.03-6.69) | (0.185-1.44) | (0.106-2.02) |
| **Xanthoma** | 1.10 | 0.441 | 2.19 |
| (95% CI) | (0.427-2.83) | (0.143-1.36) | (0.804-5.97) |
| **Enlarged fold/tortuous fold** | 1.74 | 0.599 | 0.846 |
| (95% CI) | (0.678-4.49) | (0.211-1.70) | (0.241-2.98) |
| **Nodularity** | **11.7*** | 0.950* | 0.523 |
| (95% CI) | (2.65-51.2) | (0.927-0.973) | (0.118-2.31) |
| **Regular arrangement of collecting venules (RAC)** | 0.124* | **4.64*** | **1.74*** |
| (95% CI) | (0.082-0.188) | (3.25-6.64) | (1.15-2.64) |
| **Fundic gland polyp (FGP)** | 0.312* | 1.99 | 1.52 |
| (95% CI) | (0.126-0.773) | (0.962-4.13) | (0.660-3.49) |
| **Red streak** | 0.207* | **4.04*** | 0.817 |
| (95% CI) | (0.096-0.447) | (2.22-7.36) | (0.387-1.72) |
| **Hematin** | 0.297* | **2.74*** | 1.07 |
| (95% CI) | (0.167-0.527) | (1.69-4.44) | (0.594-1.94) |
| **Raised erosion** | 0.436* | 1.01 | **2.59*** |
| (95% CI) | (0.201-0.945) | (0.503-2.01) | (1.27-5.29) |
| **Map-like redness** | 0.596 | 0.124* | **7.78*** |
| (95% CI) | (0.255-1.39) | (0.029-0.529) | (3.43-17.7) |
| **Multiple white and flat elevated lesions** | 1.37 | 1.58 | 0.996 |
| (95% CI) | (0.085-22.0) | (0.098-25.4) | (0.990-1.00) |
| **Unclear atrophy boundary (UAB)** | 0.137* | 0.622 | **7.69*** |
| (95% CI) | (0.032-0.595) | (0.238-1.63) | (3.11-19.1) |
| **RAC reappearance in atrophic mucosa**  **(RAC reappearance)** | 0.289* | 0.886 | **4.21*** |
| (95% CI) | (0.147-0.569) | (0.504-1.56) | (2.39-7.42) |

[[1]](#footnote-2)

Table 2. Diagnostic value of significant endoscopic features for current infection

|  |  | ***H. pylori* status** | | ***P***  **value** | **Sensitivity**  **(%)** | **Specificity**  **(%)** | **PPV(%)** | **NPV(%)** | **ROC/AUC** |
| --- | --- | --- | --- | --- | --- | --- | --- | --- | --- |
|  |  | **Current**  **infection** | **Other** |  | **(95%CI)** | **(95%CI)** | **(95%CI)** | **(95%CI)** | **(95%CI)** |
| **Sticky mucus** | + | 41 | 15 | <0.001 | 16.7 | 95.5 | 73.2 | 61.1 | 0.561* |
|  | - | 205 | 322 |  | (12.0-21.4) | (93.3-97.8) | (61.2-85.2) | (56.9-65.3) | (0.513-0.609) |
| **Atrophy** | + | 135 | 131 | <0.001 | 54.9 | 61.1 | 50.8 | 65.0 | 0.580* |
|  | - | 111 | 206 |  | (48.6-61.1) | (55.9-66.4) | (44.7-56.8) | (59.7-70.3) | (0.533-0.627) |
| **Diffuse redness** | + | 50 | 8 | <0.001 | 20.3 | 97.6 | 86.2 | 62.7 | 0.590* |
|  | - | 196 | 329 |  | (15.3-25.4) | (96.0-99.3) | (77.1-95.4) | (58.5-66.8) | (0.542-0.638) |
| **Spotty redness** | + | 108 | 76 | <0.001 | 43.9 | 77.4 | 58.7 | 65.4 | 0.607* |
|  | - | 138 | 261 |  | (37.7-50.1) | (73.0-81.9) | (51.5-65.9) | (60.7-70.1) | (0.560-0.654) |
| **Mucosal swelling** | + | 150 | 53 | <0.001 | 61.0 | 84.3 | 73.9 | 74.7 | 0.726* |
|  | - | 96 | 284 |  | (54.8-67.1) | (80.4-88.2) | (67.8-80.0) | (70.3-79.1) | (0.683-0.769) |
| **Nodularity** | + | 16 | 2 | <0.001 | 6.50 | 99.4 | 88.9 | 59.3 | 0.530 |
|  | - | 230 | 335 |  | (3.40-9.61) | (98.6-100) | (72.8-100) | (55.2-63.4) | (0.482-0.577) |
| **Hyperpla**  **-stic polyp** | + | 13 | 7 | 0.036 | 5.28 | 97.9 | 65.0 | 58.6 | 0.516 |
|  | - | 233 | 330 |  | (2.47-8.10) | (96.4-99.5) | (42.1-87.9) | (54.5-62.7) | (0.468-0.564) |
| **Xanthoma** | **+** | 8 | 10 | 0.844 | 3.25 | 97.0 | 44.4 | 57.9 | 0.501 |
|  | **-** | 238 | 327 |  | (1.02-5.48) | (95.2-98.9) | (19.0-69.9) | (53.8-62.0) | (0.454-0.549) |
| **Enlarged fold/tortuous fold** | **+** | 10 | 8 | 0.244 | 4.07 | 97.6 | 55.6 | 58.2 | 0.508 |
|  | **-** | 236 | 329 |  | (1.58-6.55) | (96.0-99.3) | (30.1-81.0) | (54.2-62.3) | (0.461-0.556) |
| **only one feature** | **+** | 60 | 106 | 0.062 | 24.4 | 68.5 | 36.1 | 55.4 | 0.465 |
|  | **-** | 186 | 231 |  | (19.0-29.8) | (63.6-73.5) | (28.8-43.5) | (50.6-60.2) | (0.418-0.512) |
| **one or more features** | **+** | 232 | 191 | <0.001 | 94.3 | 43.3 | 54.8 | 91.3 | 0.688* |
|  | **-** | 14 | 146 |  | (91.4-97.2) | (38.0-48.6) | (50.1-59.6) | (86.8-95.7) | (0.646-0.731) |
| **two or more features** | **+** | 172 | 85 | <0.001 | 69.9 | 74.8 | 66.9 | 77.3 | 0.723* |
|  | **-** | 74 | 252 |  | (64.1-75.7) | (70.1-79.4) | (61.1-72.7) | (72.7-81.9) | (0.681-0.766) |
| **three or more features** | **+** | 93 | 30 | <0.001 | 37.8 | 91.1 | 75.6 | 66.7 | 0.645* |
|  | **-** | 153 | 307 |  | (31.7-43.9) | (88.0-94.2) | (67.9-83.3) | (62.4-71.1) | (0.598-0.691) |

[[2]](#footnote-3)

Table 3. Diagnostic value of significant endoscopic features for no infection

|  |  | ***H. pylori* status** | | ***P* value** | **Sensitivity**  **(%)** | **Specificity**  **(%)** | **PPV(%)** | **NPV(%)** | **ROC/AUC** |
| --- | --- | --- | --- | --- | --- | --- | --- | --- | --- |
|  |  | **No infection** | **Other** |  | **(95%CI)** | **(95%CI)** | **(95%CI)** | **(95%CI)** | **(95%CI)** |
| **RAC** | + | 141 | 94 | <0.001 | 62.4 | 73.7 | 60.0 | 75.6 | 0.680* |
|  | - | 85 | 263 |  | (56.0-68.8) | (69.1-78.3) | (53.7-66.3) | (71.0-80.1) | (0.635-0.726) |
| **Red streak** | + | 38 | 17 | <0.001 | 16.8 | 95.2 | 69.1 | 64.4 | 0.560* |
|  | - | 188 | 340 |  | (11.9-21.7) | (93.0-97.5) | (56.5-81.7) | (60.3-68.5) | (0.511-0.609) |
| **Hematin** | + | 48 | 32 | <0.001 | 21.2 | 91.0 | 60.0 | 64.6 | 0.561* |
|  | - | 178 | 325 |  | (15.9-26.6) | (88.1-94.0) | (49.0-71.0) | (60.4-68.8) | (0.513-0.610) |
| **FGP** | **+** | 17 | 14 | 0.059 | 7.52 | 96.1 | 54.8 | 62.1 | 0.518 |
|  | **-** | 209 | 343 |  | (4.06-11.0) | (94.1-98.1) | (36.3-73.4) | (58.1-66.2) | (0.470-0.566) |
| **only one feature** | **+** | 122 | 106 | <0.001 | 54.0 | 70.3 | 53.5 | 70.7 | 0.621* |
|  | **-** | 104 | 251 |  | (47.4-60.5) | (65.5-75.1) | (47.0-60.0) | (66.0-75.5) | (0.574-0.669) |
| **one or more features** | **+** | 174 | 131 | <0.001 | 77.0 | 63.3 | 57.0 | 81.3 | 0.701* |
|  | **-** | 52 | 226 |  | (71.5-82.5) | (58.3-68.3) | (51.5-62.6) | (76.7-85.9) | (0.658-0.745) |
| **two or more features** | **+** | 52 | 25 | <0.001 | 23.0 | 93.0 | 67.5 | 65.6 | 0.580* |
|  | **-** | 174 | 332 |  | (17.5-28.5) | (90.3-95.7) | (56.8-78.2) | (61.5-69.8) | (0.531-0.629) |
| **three or more features** | **+** | 17 | 1 | <0.001 | 7.52 | 99.7 | 94.4 | 63.0 | 0.536 |
|  | **-** | 209 | 356 |  | (4.06-11.0) | (99.2-100) | (82.7-100) | (59.0-67.0) | (0.487-0.585) |

[[3]](#footnote-4)

**Table 4. Diagnostic value of significant endoscopic features for past infection**

|  |  | ***H. pylori* status** | | ***P*** **value** | **Sensitivity**  **(%)** | **Specificity**  **(%)** | **PPV(%)** | **NPV(%)** | **ROC/AUC** |
| --- | --- | --- | --- | --- | --- | --- | --- | --- | --- |
|  |  | **Past infection** | **other** |  | **(95%CI)** | **(95%CI)** | **(95%CI)** | **(95%CI)** | **(95%CI)** |
| **Raised erosion** | + | 13 | 23 | 0.007 | 11.7 | 95.1 | 36.1 | 82.1 | 0.534 |
|  | - | 98 | 449 |  | (5.64-17.8) | (93.2-97.1) | (19.6-52.6) | (78.9-85.3) | (0.473-0.596) |
| **Map-like redness** | + | 16 | 10 | <0.001 | 14.4 | 97.9 | 61.5 | 82.9 | 0.561* |
|  | - | 95 | 462 |  | (7.78-21.1) | (96.6-99.2) | (41.5-81.6) | (79.8-86.1) | (0.498-0.624) |
| **MWFEL** | + | 0 | 2 | 0.492 | 0 | 99.6 | 0 | 80.9 | 0.498 |
|  | - | 111 | 470 |  | 0 | (99.0-100) | 0 | (77.7-84.1) | (0.438-0.557) |
| **Atrophy** | + | 65 | 201 | 0.002 | 58.6 | 57.4 | 24.4 | 85.5 | 0.580* |
|  | - | 46 | 271 |  | (49.3-67.9) | (52.9-61.9) | (19.2-29.6) | (81.6-89.4) | (0.521-0.639) |
| **UAB** | **+** | 13 | 8 | <0.001 | 11.7 | 98.3 | 61.9 | 82.6 | 0.550 |
|  | **-** | 98 | 464 |  | (5.64-17.8) | (97.1-99.5) | (39.3-84.6) | (79.4-85.7) | (0.488-0.613) |
| **RAC reappearance** | **+** | 26 | 32 | <0.001 | 23.4 | 93.2 | 44.8 | 83.8 | 0.583* |
|  | **-** | 85 | 440 |  | (15.4-31.4) | (90.9-95.5) | (31.6-58.0) | (80.6-87.0) | (0.520-0.646) |
| **only one feature** | **+** | 36 | 166 | 0.586 | 32.4 | 64.8 | 17.8 | 80.3 | 0.486 |
|  | **-** | 75 | 306 |  | (23.6-41.3) | (60.5-69.2) | (12.5-23.1) | (76.3-84.3) | (0.427-0.546) |
| **one or more features** | **+** | 74 | 217 | <0.001 | 66.7 | 54.0 | 25.4 | 87.3 | 0.603* |
|  | **-** | 37 | 255 |  | (57.8-75.6) | (49.5-58.5) | (20.4-30.5) | (83.5-91.2) | (0.546-0.661) |
| **two or more features** | **+** | 38 | 51 | <0.001 | 34.2 | 89.2 | 42.7 | 85.2 | 0.617* |
|  | **-** | 73 | 421 |  | (25.3-43.2) | (86.4-92.0) | (32.2-53.2) | (82.1-88.4) | (0.554-0.680) |
| **three or more features** | **+** | 19 | 7 | <0.001 | 17.1 | 98.5 | 73.1 | 83.5 | 0.578* |
|  | **-** | 92 | 465 |  | (10.0-24.2) | (97.4-99.6) | (54.8-91.3) | (80.4-86.6) | (0.515-0.642) |

[[4]](#footnote-5)

1. **P*<0.05 [↑](#footnote-ref-2)
2. Other: No infection and Past infection

   +: present; -: absent. NPV: negative predictive value; PPV: positive predictive value; ROC/AUC: area under the curve of receiver operating characteristics; **P* < 0.05 (ROC/AUC) [↑](#footnote-ref-3)
3. Other: Current infection and Past infection

   RAC: Regular arrangement of collecting venules ; FGP: Fundic gland polyp

   +: present; -: absent. NPV: negative predictive value; PPV: positive predictive value; ROC/AUC: area under the curve of receiver operating characteristics; **P* < 0.05 (ROC/AUC) [↑](#footnote-ref-4)
4. Other: Current infection and No infection

   MWFEL: Multiple white and flat elevated lesions; UAB: Unclear atrophy boundary; RAC reappearance: RAC reappearance in atrophic mucosa

   +: present; -: absent. NPV: negative predictive value; PPV: positive predictive value; ROC/AUC: area under the curve of receiver operating characteristics; **P* < 0.05 (ROC/AUC) [↑](#footnote-ref-5)
